# ARID1A deficiency identifies molecular alterations and potential therapeutic implications in cervical clear cell carcinoma: an integrated genomic and clinicopathological study

**DOI:** 10.3389/fonc.2026.1943760

**Published:** 2026-09-21

**Authors:** Xiaoni Zhong, Chao Wang, Hongying Zhang, Huirong Zhang, Yibo Fan, Hongtao Jin, Kaipeng Huang, Jianrong Huang, Xiaoshi Ma, Xiaojing Guo, Xiaoyan Wu

**Affiliations:** 1 Department of Pathology, Shenzhen People’s Hospital, The Second Clinical Medical College of Jinan University, The First Affiliated Hospital of Southern University of Science and Technology, Shenzhen, Guangdong, China; 2 Mental Rehabilitation Department, Shenzhen Mental Health Center, Shenzhen Kangning Hospital, Shenzhen, Guangdong, China; 3 Department of Pathology, Shenzhen Third People’s Hospital, The Second Affiliated Hospital of Southern University of Science and Technology, Shenzhen, Guangdong, China; 4 Clinical Research Center for Rare Diseases, The University of Hong Kong-Shenzhen Hospital, Shenzhen, Guangdong, China; 5 Department of Nephrology, Shenzhen Third People’s Hospital, The Second Affiliated Hospital of Southern University of Science and Technology, Shenzhen, Guangdong, China; 6 Department of Molecular Diagnostics, State Key Laboratory of Oncology in South China, Guangdong Provincial Clinical Research Center for Cancer, Sun Yat-sen University Cancer Center, Guangzhou, Guangdong, China

**Keywords:** ARID1A deficiency, cervical clear cell carcinoma, precision oncology, SWI/SNF chromatin remodeling complex, therapeutic vulnerability

## Abstract

**Background:**

Cervical clear cell carcinoma (CCC) is a rare and aggressive subtype of cervical adenocarcinoma whose molecular drivers and therapeutic vulnerabilities remain incompletely defined. As a key component of the SWI/SNF chromatin-remodeling complex, ARID1A has been recognized as a potential biomarker of therapeutic response across multiple human malignancies. Nevertheless, the molecular and clinical significance of ARID1A in CCC has not been systematically clarified. This study aimed to characterize the genomic landscape of CCC and explore whether ARID1A deficiency serves as a molecular signature that confers actionable therapeutic vulnerabilities for precision oncology.

**Methods:**

We retrospectively collected clinicopathological data from 27 patients diagnosed with CCC at Sun Yat-sen University Cancer Center between August 2018 and May 2023. Targeted next-generation sequencing (NGS) was performed using a cancer-related panel with complete exon coverage of 312 genes and selected-region coverage of additional cancer-associated genes in tumor tissues and matched peripheral blood samples from 7 patients to identify recurrent genomic alterations and perturbed biological pathways. Functional enrichment analyses, including Gene Ontology (GO), Kyoto Encyclopedia of Genes and Genomes (KEGG), and protein–protein interaction (PPI) network analyses, were conducted to identify altered pathways and candidate hub genes. Based on the integrated genomic and network analyses, ARID1A was selected for further evaluation, and ARID1A protein expression was evaluated by immunohistochemistry in 27 CCC samples and 30 HPV-associated cervical adenocarcinoma (HPVA) samples.

**Results:**

The median age of enrolled patients was 47.6 years, and 74.1% of patients presented with early-stage disease (FIGO IA–IIA). With a median follow-up duration of 44.6 months, the 3- and 5-year overall survival rates were 90% and 77%, respectively. Targeted NGS identified a total of 93 somatic alterations spanning 78 genes, with recurrent alterations predominantly occurring in ARID1A, PIK3CA, SMARCA4, and JAK3. Pathway enrichment revealed significant perturbations in chromatin-remodeling, PI3K-AKT, p53, and JAK-STAT signaling pathways. PPI network analysis identified ARID1A, PIK3CA, EGFR, and CTNNB1 as core hub molecules mediating tumorigenesis. Complete loss of nuclear ARID1A expression was detected in 51.8% of CCC cases, significantly higher than the 6.0% rate detected in HPVA lesions (*P* < 0.001).

**Conclusion:**

CCC exhibits a distinct molecular profile characterized by frequent disruption of chromatin-remodeling pathways. Enrichment analyses linked ARID1A deficiency to concurrent genomic aberrations affecting chromatin remodeling and PI3K/AKT cascades. Our findings suggest that ARID1A loss could act as a potential adjunctive diagnostic marker when interpreted together with histopathological features, HPV status, and other immunohistochemical markers, while representing a therapeutic vulnerability requiring further validation.

## 1 Introduction

In 2018, the International Endocervical Adenocarcinoma Criteria and Classification (IECC) system established a pathogenetic taxonomy of cervical adenocarcinomas, formally distinguishing human papillomavirus (HPV)-associated and non-HPV-associated subtypes (1). As a prototypical non-HPV-driven entity, cervical clear cell carcinoma (CCC) accounts for approximately 4% to 9% of cervical adenocarcinomas and exhibits inherently aggressive biological behavior (2). Distinct from HPV-related cervical malignancies, CCC lacks consistent HPV infection and bears unique morphological and molecular signatures. Early retrospective cohorts published in 1971 linked to diethylstilbestrol (DES) expose to increased risk of clear cell carcinoma of the female genital tract (3). DES was withdrawn from clinical practice following the identification of its carcinogenic potential, and most contemporary CCC cases arise sporadically without historical DES exposure (4).

Clinically, CCC often presents overlapping morphological features with other cervical malignancies at initial diagnosis, yet it preferentially grows endophytically and demonstrates greater propensity for uterine corpus extension compared with squamous cell carcinoma (SCC) and HPV-associated adenocarcinomas (HPVA) (5). Mounting clinical evidence indicates that CCC confers inferior prognostic outcomes compared with HPV-associated adenocarcinomas, as reflected by reduced 5-year and 10-year overall survival (OS) rates (6). These clinical disparities highlight the urgent demand for improved diagnostic and therapeutic strategies for CCC. Although endometriosis has been proposed as a potential precursor lesion for a subset of CCC cases, the etiology of this rare cancer remains largely elusive. Prior molecular studies have implicated dysregulation of oncogenic pathways, including aberrant Bcl-2 expression and p53 alterations, in CCC pathogenesis (7, 8). Additionally, the PIK3CA-AKT-mTOR signaling cascade is frequently activated in CCC, evidenced by elevated phosphorylated AKT (p-AKT) and phosphorylated mTOR (p-mTOR), which jointly sustain tumor proliferation and cell survival (9). Nevertheless, the comprehensive molecular landscape driving CCC initiation and progression remains incompletely understood.

The SWI/SNF chromatin-remodeling complex regulates gene expression, DNA replication, and DNA repair through ATP-dependent nucleosome remodeling. ARID1A, a key tumor-suppressive subunit of this complex, contributes to its regulatory specificity (10). Loss of ARID1A expression has been reported in several gynecologic malignancies, accompanied by distinct molecular and clinicopathological characteristics. Nonetheless, the biological and clinical significance of ARID1A deficiency in CCC remains poorly understood (11, 12).

Recent advances in next-generation sequencing (NGS) and computational bioinformatics have enabled comprehensive characterization of cancer genomes to identify driver molecular events and therapeutic targets. Integrative multi-omics analyses have identified disrupted PI3K/AKT signaling, Wnt/β-catenin signaling, cell-cycle regulation, and chromatin-remodeling pathways as key drivers of cervical carcinogenesis. Moreover, network-based approaches, including PPI and pathway enrichment analyses, provide valuable strategies for identifying central regulatory genes and potential therapeutic vulnerabilities. Despite these technical innovations, few studies have integrated genomic profiling, protein-level validation and clinical interpretation to systematically dissect CCC molecular features.

Among the recurrent genomic alterations identified in CCC, ARID1A was prioritized for in-depth investigation because it encodes an indispensable SWI/SNF subunit with well-established tumor-suppressive functions across multiple cancer types. In addition, ARID1A deficiency can be evaluated at the protein level by immunohistochemistry, enabling integration of genomic findings with routine pathological assessment. Furthermore, previous research across other cancer types has linked ARID1A deficiency to actionable therapeutic vulnerabilities, providing a strong rationale for exploring its translational value in CCC.

In this study, we performed targeted NGS and ARID1A immunohistochemical profiling to characterize the molecular landscape of CCC. We systematically compared ARID1A protein loss frequency between CCC and HPVA, characterized perturbed signaling pathways enriched among mutated genes, and interrogated the biological and therapeutic implications of ARID1A deficiency in this rare tumor. Our integrated multi-layered analysis provides evidence supporting ARID1A as an auxiliary diagnostic marker and a candidate therapeutic biomarker to guide future precision treatment development for CCC.

## 2 Materials and methods

### 2.1 Sample collections

Clinical data of patients histologically diagnosed with CCC at Sun Yat-sen University Cancer Center, Guangzhou, Guangdong Province from August 2018 to May 2023 were extracted from the electronic medical record system. This data included age, initial symptoms, and HPV status. Clinicopathological characteristics encompassed tumor size, histopathological morphology, depth of cervical stromal invasion, lymph node metastasis status, and parametrial involvement. Overall survival (OS) was defined as the period from treatment initiation to either disease-related death or the last follow-up date. Progression-free survival (PFS) was measured from the treatment date to the recurrence or radiological progression of the disease.

A total of 27 paraffin samples from patients with CCC and 30 paraffin samples from patients with HPV-related cervical adenocarcinoma (HPVA) were collected. Patients were staged based on the 2018 FIGO guidelines. Histopathological diagnoses of all cases were independently re-reviewed by two experienced gynecologic pathologists. The diagnoses were confirmed based on morphological characteristics and relevant immunohistochemical findings, and any discrepancies were resolved through consensus review.

All cases included in the present study were obtained from a single institution, and no independent external cohort was available for validation. Accordingly, the diagnostic performance of ARID1A loss observed in this cohort should be considered exploratory and requires verification in larger multicenter cohorts. The study adhered to the World Medical Association’s Declaration of Helsinki ethical guidelines and was approved by the Cancer Center of Sun Yat-sen University (Approval number B2020-344-01). Written informed consent was obtained from all participants before sample collection.

### 2.2 Targeted NGS

Seven CCC cases with sufficient tumor tissue volume and adequate DNA quality were selected for targeted NGS. Case inclusion was determined by sample availability and sequencing quality requirements rather than random sampling to avoid inherent selection bias. Therefore, all genomic findings derived from this small subgroup are exploratory and warrant external validation in larger cohorts.

The paraffin-embedded tissues were sliced continuously, and DNA was extracted using the nucleic acid automatic extractor along with supporting reagents from Keshuo Biotechnology (Xiamen) Co., Ltd. DNA library was constructed and DNA nano-spheres (DNB) were prepared using reagents provided by Suzhou GenePlus Biomedical Engineering Co., Ltd., followed by sequencing on the gene+seq-2000 gene sequencer. The custom sequencing panel contained three independent capture modules (**Supplementary Table 1**): (1) full exons of 312 core cancer-driving genes; (2) intronic and promoter fusion breakpoint regions of 38 recurrent fusion-associated genes; (3) partial hotspot exon regions of additional 709 cancer-related genes. Sequencing achieved a mean coverage depth of at least 500× for tumor samples and at least 100× for matched blood controls.

Variant calling followed GATK best practices, with Sanger validation of pathogenic mutations. Sensitivity and specificity were confirmed using the GIAB reference standard (HG001). Raw sequencing data undergoes base calling, sequence alignment, variation identification, and variation annotation for data analysis. All variants were named according to the guidelines established by the Human Genome Variation Society (HGVS), with genomic coordinates aligned to the GRCh37 human reference assembly. Somatic variants were classified according to AMP/ASCO/CAP guidelines and annotated using ClinVar, COSMIC, and OncoKB databases to evaluate their potential oncogenic significance and therapeutic relevance.

### 2.3 Functional enrichment and PPI network analysis

We utilized the Phyper function for hypergeometric test-based Gene Ontology (GO) and Kyoto Encyclopedia of Genes and Genomes (KEGG) enrichment analyses to explore the functions of variant genes. Pathways with significant enrichment were identified using an unadjusted *P-*value threshold of <0.05. Multiple-testing correction was not performed given the exploratory nature and limited input gene number of this analysis. Protein–protein interaction (PPI) networks of variant genes were conducted using the STRING database. A threshold of a comprehensive score exceeding 0.9 was set, and the outcomes were visualized with Cytoscape. The MCODE plugin in Cytoscape was applied to identify modules within the PPI network, and the Degree algorithm was utilized to pinpoint hub genes.

### 2.4 ARID1A immunohistochemistry

Formalin-fixed paraffin-embedded (FFPE) tissue sections were cut at 4 μm thickness, followed by deparaffinization and hematoxylin and eosin (H&E) staining to identify representative tumor areas. Immunohistochemical staining (IHC) was performed using an anti-ARID1A antibody (dilution 1:100; JJ09-01; SAB, USA) with the EnVision detection system and diaminobenzidine (DAB) as the chromogenic substrate. ARID1A immunoreactivity was evaluated based on nuclear staining signals in tumor cells. Nuclear staining in tumor cells was considered positive when unequivocal nuclear expression was observed with appropriate internal positive controls. ARID1A loss was defined as complete absence of nuclear ARID1A staining in tumor cells with preserved staining in internal positive control, including adjacent non-neoplastic epithelial cells, stromal cells, inflammatory cells, or fibroblasts. Cases showing any retained nuclear staining in tumor cells were classified as ARID1A-positive, whereas cases with complete loss of tumor nuclear staining were classified as ARID1A-negative. No semi-quantitative scoring system or percentage threshold was applied, as the analysis focused on complete loss of ARID1A protein expression. Two experienced pathologists blinded to all clinical metadata independently evaluated all IHC slides, with consensus reached for discrepant cases.

### 2.5 Statistical analysis

All statistical analyses were executed using SPSS version 27.0. The Kaplan–Meier method was used to estimate PFS and OS, and inter-group survival differences were compared using the log-rank test. Univariate Cox proportional hazards regression analyses were performed to estimate hazard ratios. Given the limited number of events and the small cohort size, survival analyses were considered exploratory, and effect estimates were interpreted with extreme caution due to potential statistical instability. Descriptive statistics were used to summarize clinical outcomes. Small sample sizes (N = 7 for NGS) limited statistical power for subtype analyses. Statistical significance was defined as a *P*-value below 0.05.

## 3 Results

### 3.1 Baseline clinicopathological characteristics and survival outcomes

This retrospective cohort enrolled 27 patients with histologically confirmed CCC (**Figure 1**). Patients age ranged from 26 to 79 years, with an average age a median age of 47 years (mean: 47.55 ± 12.20 years). All patients received radical hysterectomy plus bilateral adnexectomy and pelvic lymph node dissection. Among them, three patients underwent additional para-aortic lymph node resection, while one patient had an additional right para-aortic lymph node biopsy. In other three cases, omentectomy, resection of lesions surrounding the rectum, and pelvic adhesion lysis were performed. Postoperative platinum-taxane chemoradiotherapy was administered to patients with high-risk pathological features, including lymph vascular invasion and lymph node metastasis. Tumor cut surfaces presented grey-white or gray-red tissue with variable texture (**Figure 2A**). Microscopically, tumors formed papillary, cribriform, or tubular patterns, with hobnail-shaped tumor cells containing clear cytoplasm and markedly atypical hyperchromatic nuclei (**Figure 2B**). In total, 74.1% of patients were diagnosed with early-stage CCC (FIGO IA–IIA), while the remaining 25.9% presented advanced-stage disease (IIB–IV).

**Figure 1:**
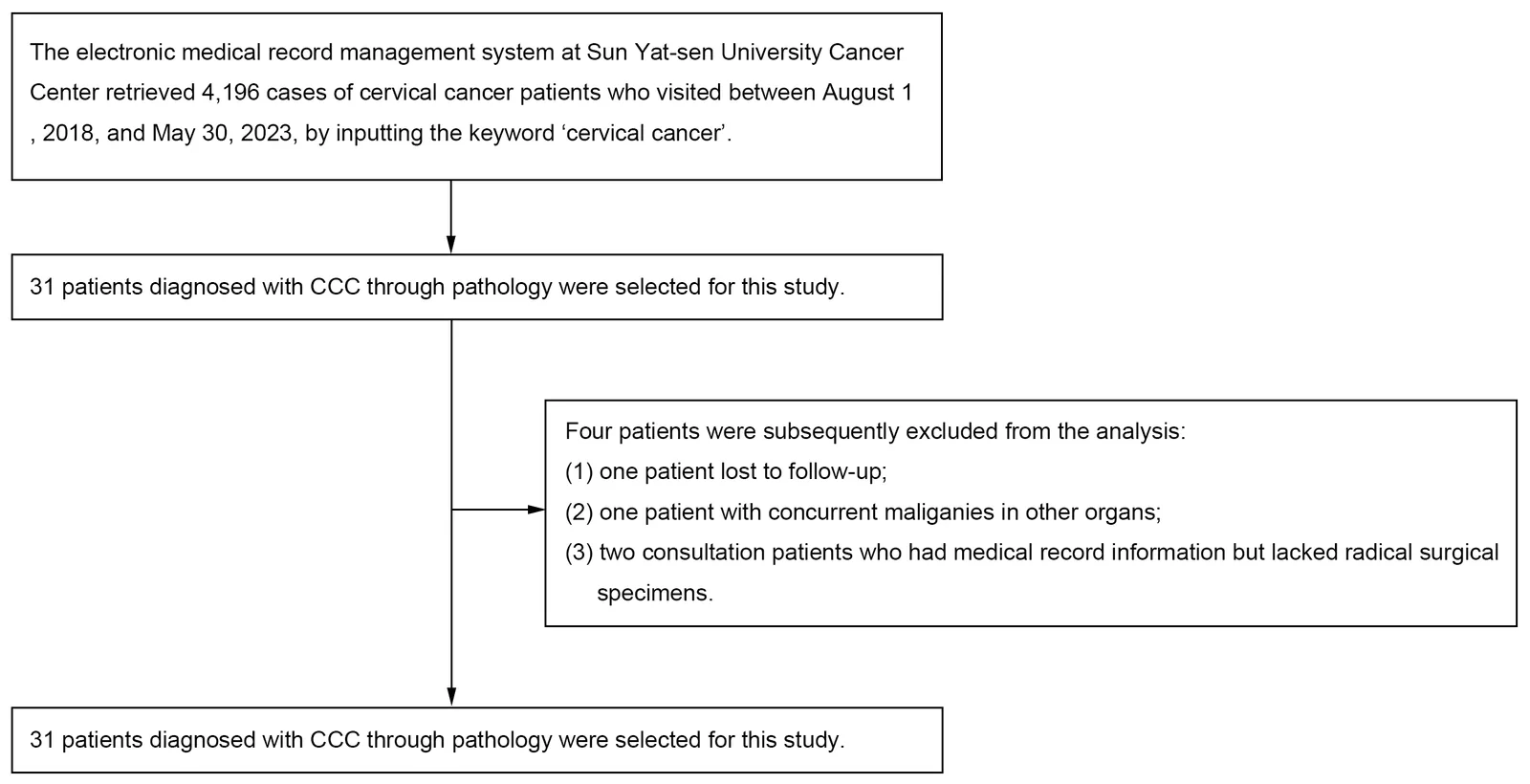
Flow chart of CCC patient screening.

**Figure 2:**
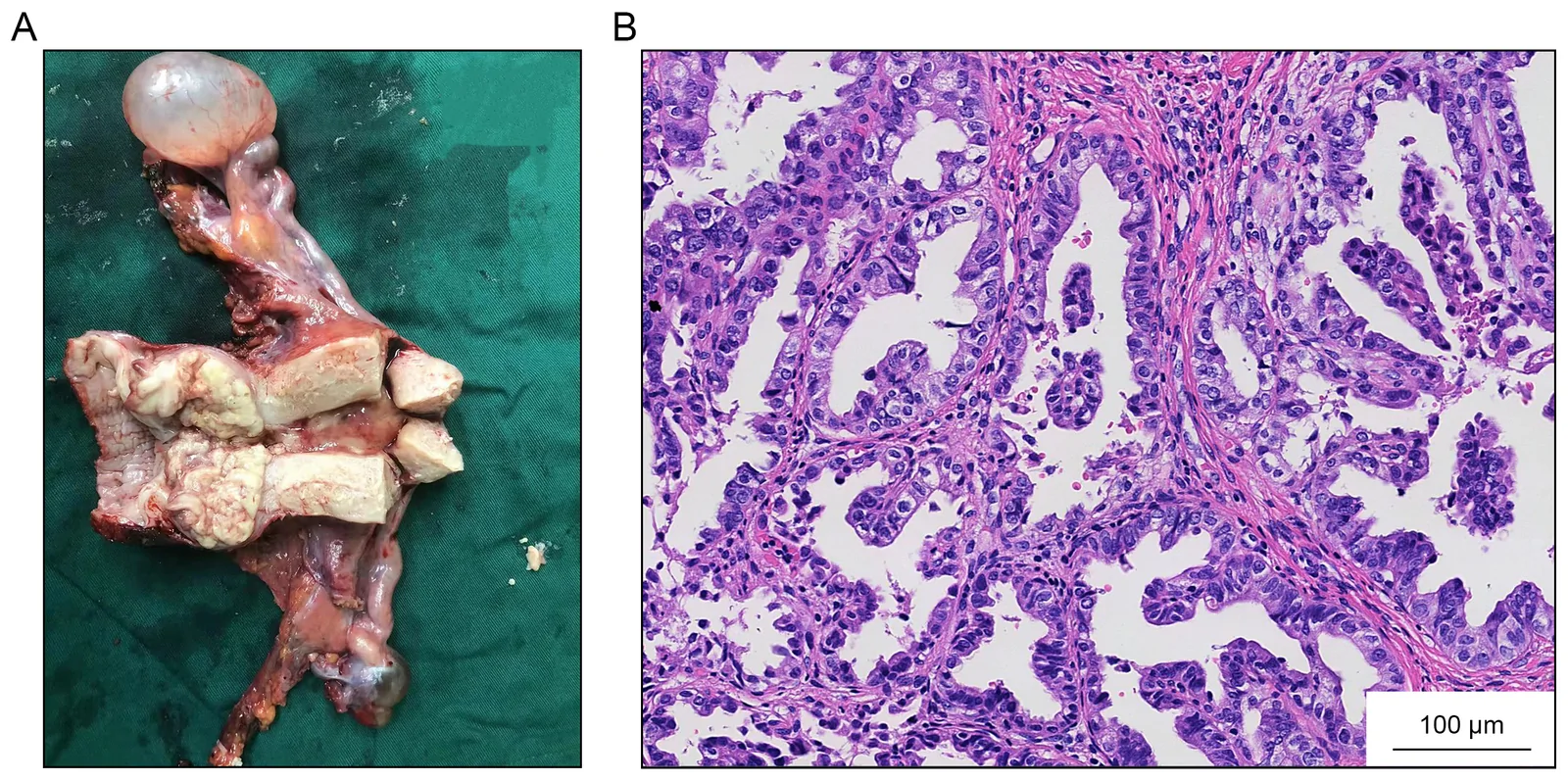
The macroscopic and microscopic hematoxylin-eosin (HE) morphology of the entire uterus in patients with CCC. Magnification ×200. **(A)** The cut surface of the tumor exhibits a firm texture with gray-white to gray-yellow coloration; **(B)** The cancer tissue is arranged in a papillary and glandular tubular pattern. The tumor cells exhibit transparent or eosinophilic cytoplasm, with some cells organized in a nail-like pattern. The nuclei are large, deeply stained, and exhibit significant atypia.

The median follow-up duration reached 44.62 months (ranged 16–71 months). The 3-year and the 5-year survival rates were 90% and 77%, respectively. Five patients died of tumor-related disease, and 6 patients experienced recurrence or metastasis after surgery. Kaplan–Meier survival analysis with log-rank testing identified tumor size, FIGO stage (IIB–IV), lymph node metastasis, vaginal tissue infiltration, and postoperative adverse recurrence as factors associated with shorten PFS (*P* < 0.05, **Table 1**). Univariate Cox regression further confirmed lymph node metastasis (HR = 0.227, 95% CI 0.076–0.682, *P* = 0.008) and vaginal tissue infiltration (HR = 0.371, 95% CI 0.139–0.992, *P* = 0.048) as significant poor prognostic indicators. Conversely, other clinical variables lost statistical significance in Cox models, likely due to limited survival events and wide confidence intervals.

**Table 1:** Clinical pathological features and univariate analysis (Log-rank test) of 27 patients with CCC.

Clinical characteristics	Number of cases	Percentage (%)	χ²	*P*
Age			1.57	0.210
≥40 years old	21	74.1		
<40 years old	6	25.9		
Tumor Diameter			10.02	0.002
≥4cm	11	40.7		
<4 cm	16	59.2		
FIGO Staging			23.75	<0.001
IA- IIA	21	77.8		
IIB-IV	6	22.2		
Depth of Cervical Stromal Invasion			2.06	0.152
>2/3 of the fibromuscular layer	13	48.1		
≤2/3 of the fibromuscular layer	14	51.9		
Vascular Tumor Thrombus			0.77	0.378
Present	3	11.1		
Absent	24	88.9		
Lymph Node Metastasis			13.63	<0.001
Present	6	22.2		
Absent	21	77.8		
Involvement of Vaginal Tissue			5.34	0.021
Present	3	11.1		
Absent	24	88.9		
Recurrence and Metastasis			21.37	<0.001
Present	6	22.2		
Absent	21	77.8		

Due to the limited number of survival events, Cox regression analyses were considered exploratory and were not intended for definitive prognostic modeling. CCC, cervical clear cell carcinoma; FIGO, International Federation of Gynecology and Obstetrics. Survival comparisons were performed using Kaplan–Meier analysis with log-rank testing.

Exploratory univariate Cox regression analysis was performed to estimate the association between clinicopathological factors and PFS. Although lymph node metastasis and vaginal tissue infiltration showed associations with PFS in this analysis, the estimates demonstrated wide confidence intervals, reflecting the limited number of events and small sample size. Therefore, these findings should be interpreted as hypothesis-generating rather than definitive prognostic associations. Several clinicopathological variables showed differences in univariate survival analyses; however, Cox regression estimates were unstable because of the limited number of outcome events and should therefore be interpreted cautiously.

### 3.2 Genomic profiling identifies recurrent somatic alterations and perturbed therapeutic pathways

Targeted NGS was performed on pair tumor and peripheral blood samples from seven CCC patients to investigate the molecular changes linked to CCC. A total of 93 somatic variants across 78 genes were identified, including 41 missense mutations, 4 nonsense mutations, 20 frame-shift indels, 6 deletions, 4 intron splicing mutations, 2 copy number amplifications, and 1 gene fusion (**Figure 3**). The number of detected somatic alterations varied among individual CCC samples, with a median of 5 variants per sample (range: 2–64 alterations/sample), indicating heterogeneity in genomic alteration patterns among the sequenced cases (**Supplementary Table 2**).

**Figure 3:**
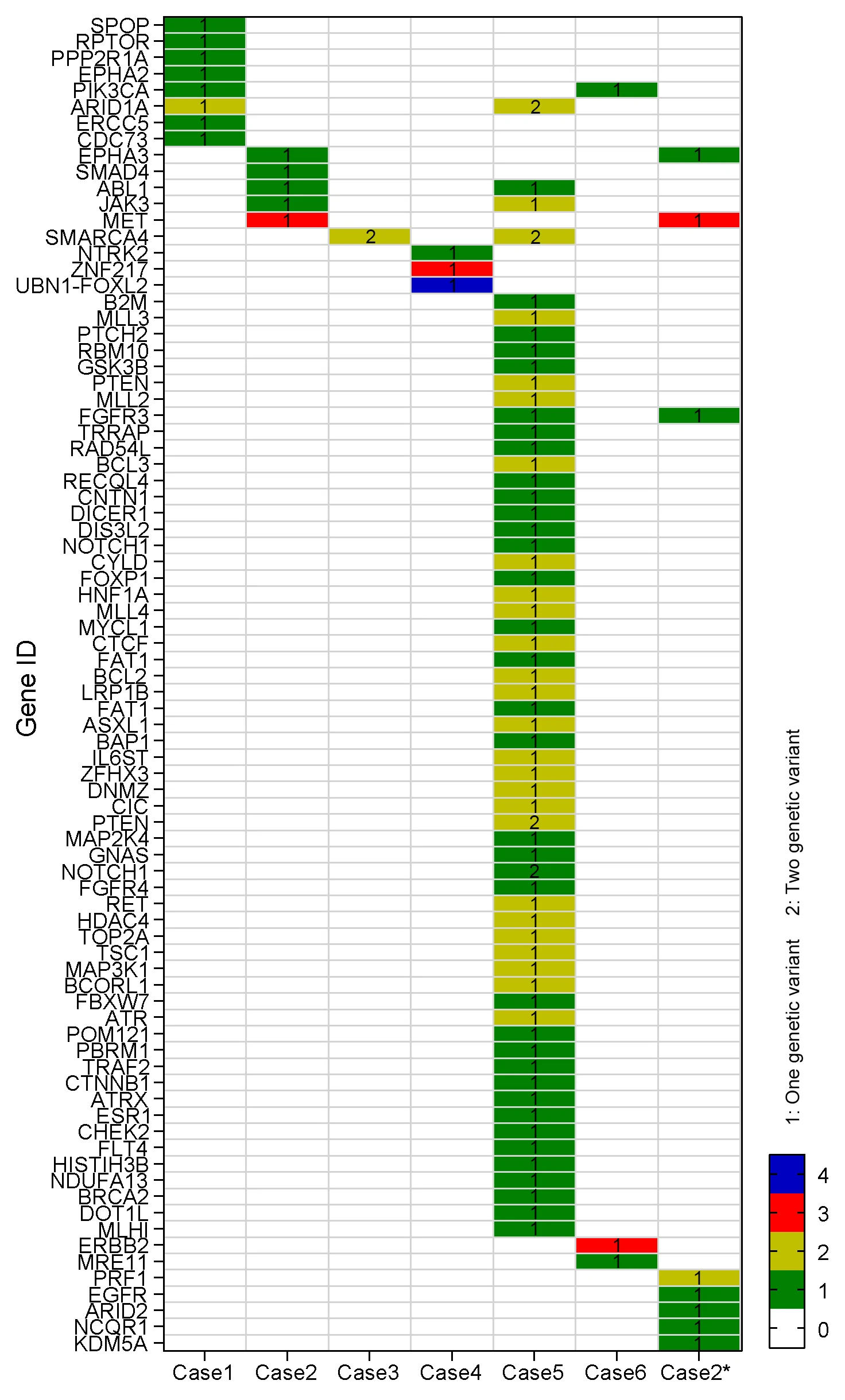
Genetic mutation landscape of seven CCC samples. Green indicates point mutations, yellow represents small fragment insertions or deletions, red denotes copy number variations, and blue illustrates gene rearrangements.

Recurrently mutated genes (≥2 variants) included PIK3CA, ARID1A, EPHA3, ABL1, JAK3, SMARCA4, FGFR3, MET, and NOTCH1. Three patients exhibited concurrent alterations in chromatin remodeling genes such as SMARCA4, ARID1A and ARID2, highlighting frequent epigenetic pathway disruption in CCC. One patient with a pathogenic germline MLHI germline variant (NM_000249.3: c.199G>A, p.G67R) consistent with Lynch syndrome criteria, no extra-cervical tumors or relevant family history. GO and KEGG enrichment analyses were performed to annotate functional pathways perturbed by mutated genes. Cellular components (CC) terms centered on chromatin structure and epigenetic modification (**Figure 4A**). Biological processes were enriched in chromatin remodeling, peptidyl-tyrosine phosphorylation, positive regulation of the ERK1/ERK2 cascade (**Figure 4B**). Molecular functions mainly involved in protein binding, transmembrane receptor protein tyrosine kinase activity, and protein tyrosine kinase activity (**Figure 4C**). Subsequent KEGG enrichment analysis demonstrated predominant association with PI3K-Akt, AMPK, p53, JAK-STAT, and WNT signaling pathways (**Figure 4D**). These computational enrichments suggest a statistical link between somatic variants and core oncogenic cascades, without direct functional verification at protein level.

**Figure 4:**
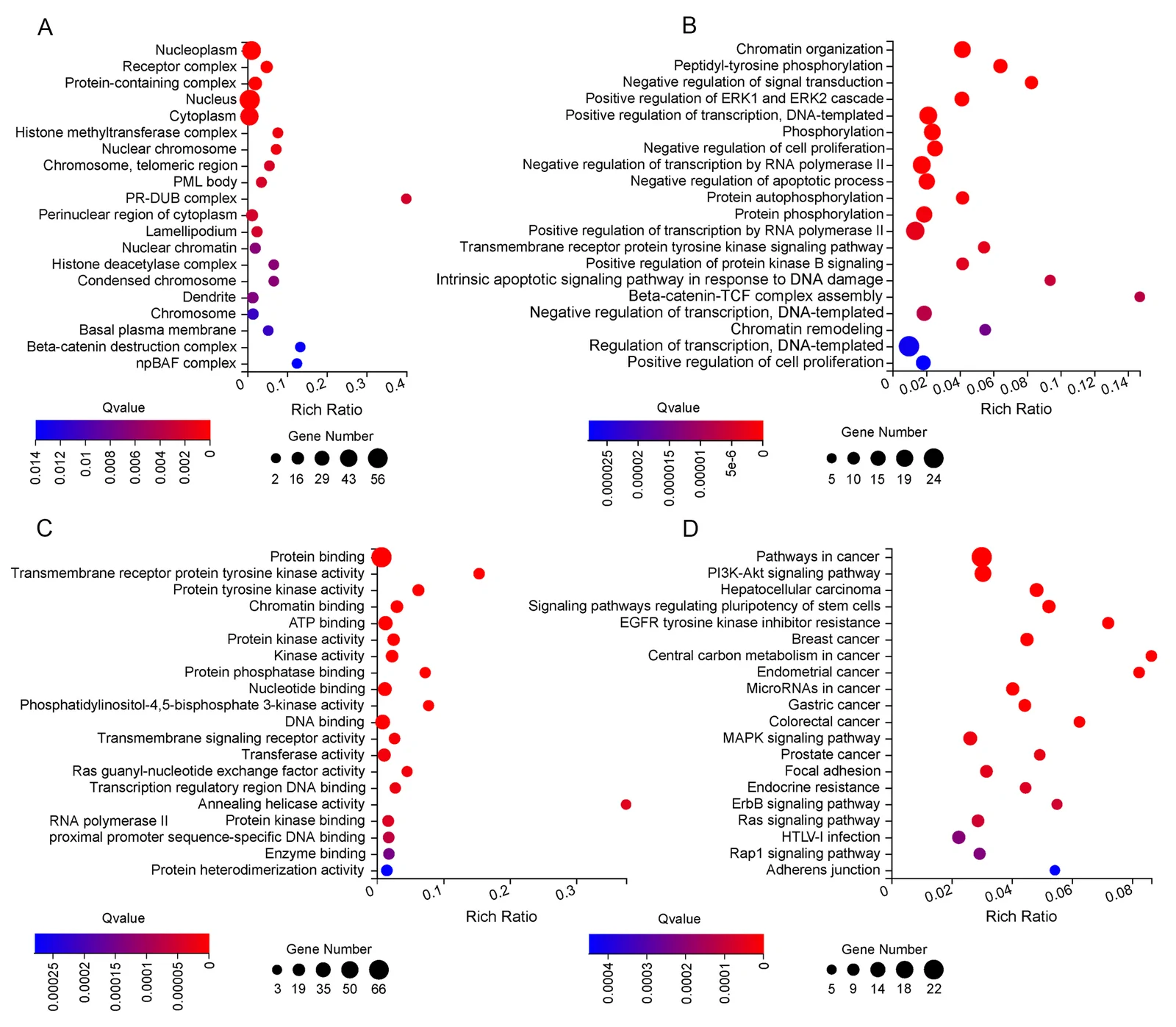
Presents the GO and KEGG enrichment analyses of mutant genes. **(A)** Cellular Component (CC) analysis; **(B)** Biological Process (BP) analysis; **(C)** Molecular Function (MF) analysis; **(D)** KEGG functional enrichment analysis. All for the mutant genes.

### 3.3 PPI network dissects hub genes and therapeutic functional modules

The functional network of mutant genes was further analyzed by constructing a PPI network with a confidence level of 0.9 using the STRING database and visualized with Cytoscape (**Figure 5A**). The degree algorithm was employed to rank the proteins within the PPI network and the top 10 genes were CTNNB1, PIK3CA, EGFR, PTEN, ESR1, ERBB2, ARID1A, JAK3, SMARCA4, and H3C12 (**Figure 5B**). Among these hub genes, ARID1A was selected for further investigation because it was recurrently altered in the CCC sequencing cohort and encodes a key subunit of the SWI/SNF chromatin-remodeling complex with established tumor suppressor functions. In addition, unlike several other hub genes identified through network analysis, ARID1A protein expression can be directly evaluated by immunohistochemistry, enabling integrated genomic and protein-level validation in CCC samples.

**Figure 5:**
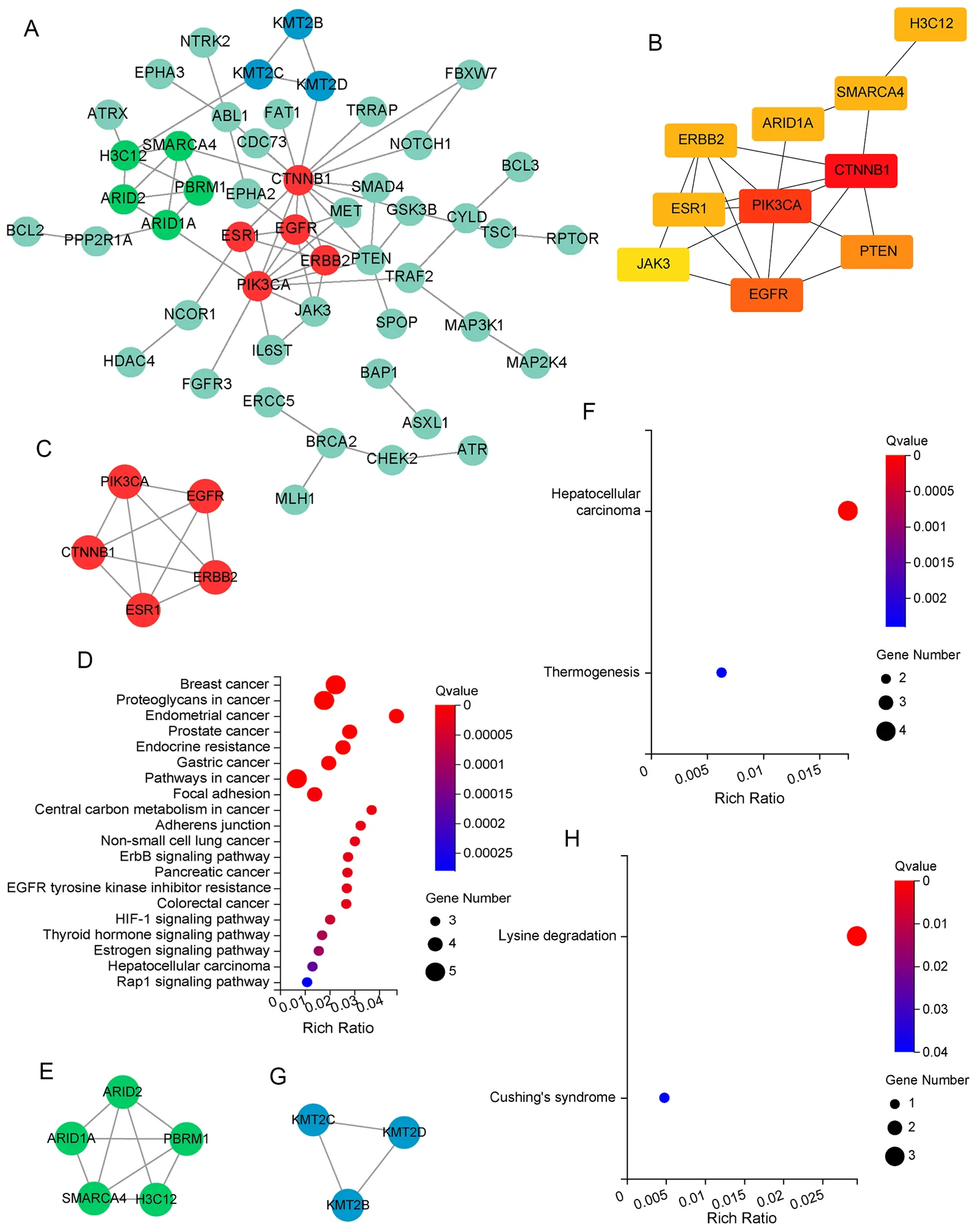
PPI network analysis of mutant genes. **(A)** The PPI network for all mutant genes. **(B)** The top 10 genes ranked by degree. **(C, E, G)** MCODE1–3 modules identified from the PPI network. **(D, F, H)** KEGG analyses of the genes corresponding to MCODE1–3 modules. PPI represents protein–protein interaction analysis. MCODE (Molecular Complex Detection) was used to identify densely connected modules within the PPI network.

Three tightly connected functional modules were extracted using the MCODE plugin. Module 1 contained EGFR, PIK3CA, ESR1, ERBB2 and CTNNB1, associated with the ErbB and HIF-1 signaling pathway (**Figures 5C, D**). Module 2 consisted of SWI/SNF complex subunits PBRM1, SMARCA4, H3C12, ARID2 and ARID1A, linked to thermogenesis pathways (**Figures 5E, F**). Module 3 comprised histone modification genes, KMT2C, KMT2D and KMT2B, enriched in lysine degradation (**Figures 5G, H**). The modular network structure further emphasizes the central role of chromatin remodeling and PI3K/AKT signaling in CCC pathogenesis.

### 3.4 ARID1A protein loss is a distinct molecular signature and therapeutic vulnerability in CCC

ARID1A physiological expression presented as diffuse brown nuclear staining in tumor epithelial cells, while ARID1A-deficient cases exhibited complete absence of tumor nuclear immunoreactivity with intact staining in internal stromal and inflammatory control cells (**Figure 6**). Among the 27 CCC tissue samples, 14 cases (51.8%) exhibited full ARID1A protein loss in tumor tissues. In contrast, only 2 out of 30 HPVA control cases (6.7%) displayed ARID1A deficiency, representing a highly significant difference between the two histological subtypes (*P* < 0.001, **Table 2**).

**Figure 6:**
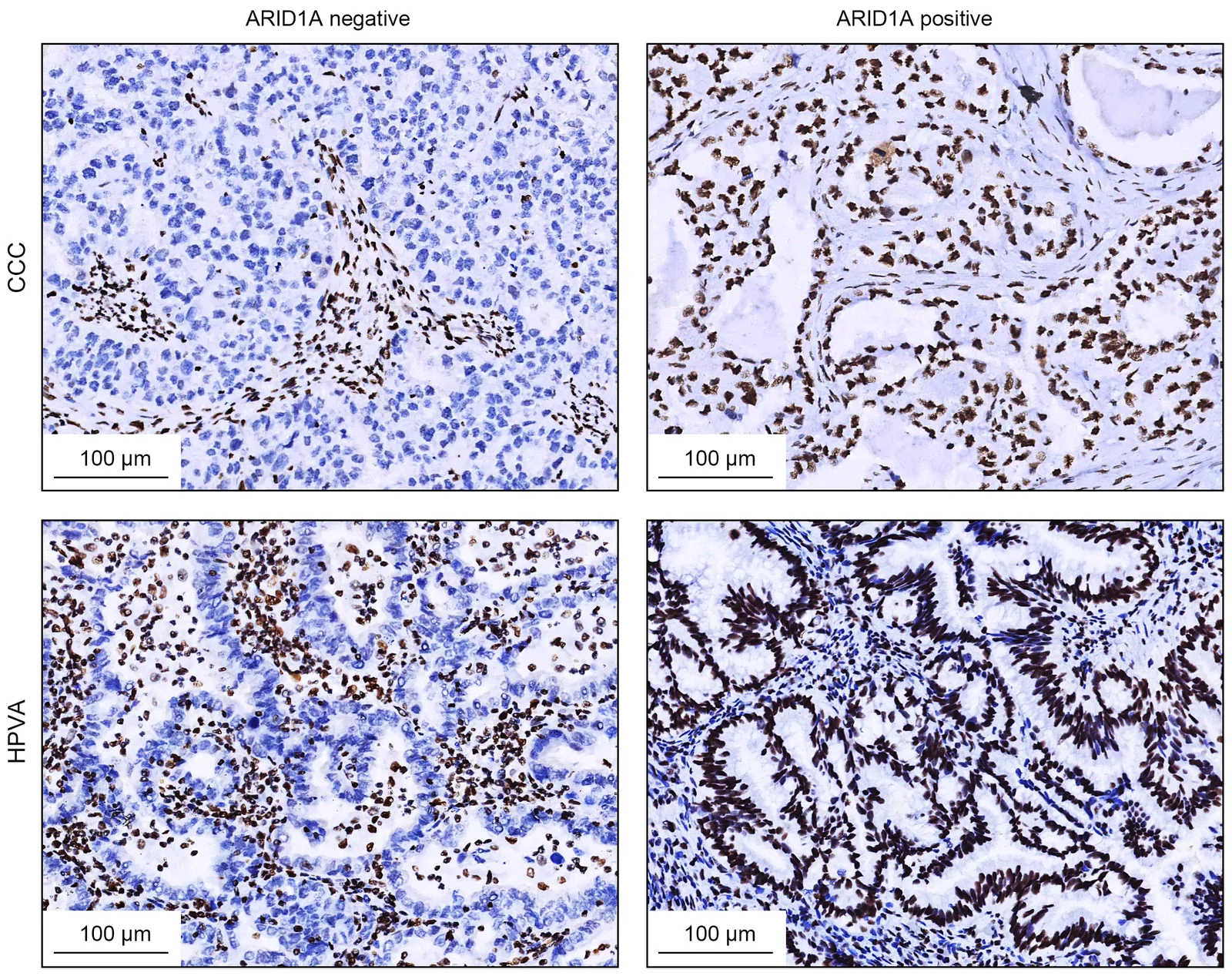
Representative ARID1A immunohistochemical staining patterns in CCC and HPVA tissues (×200 magnification). ARID1A-positive cases show diffuse or retained brown nuclear staining in tumor cells, whereas ARID1A-negative cases demonstrate complete loss of tumor cell nuclear staining with preserved internal positive controls. ARID1A loss was defined as the absence of nuclear staining in tumor cells despite retained nuclear staining in non-neoplastic internal control cells.

**Table 2:** The expression status of ARID1A in CCC and HPV-related cervical adenocarcinoma (HPVA) tissues.

Type of tissue	Number of cases	ARID1A not expressed, n (%)	ARID1A expressed, n (%)	*P* value
CCC	27	14 (51.8%)	13 (48.2%)	<0.001
HPVA	30	2 (6.6%)	28 (93.4%)	

CCC, cervical clear cell carcinoma; HPVA, human papillomavirus-associated cervical adenocarcinoma.

Within this single-institution cohort, ARID1A loss achieved 51.8% sensitivity and 93.3% specificity for differentiating CCC from HPVA. The binary evaluation standard relying on complete nuclear loss eliminates complex semi-quantitative scoring systems, lowering inter-observer variation and facilitating routine pathological diagnosis. Previous independent sequencing work has reported ARID1A somatic mutations in 20% of CCC cases, yet no prior research quantified ARID1A protein loss via IHC and compared its diagnostic efficiency with HPVA. Our IHC results fill this research gap and provide straightforward morphological auxiliary evidence for pathologists to distinguish rare CCC from common HPV-driven adenocarcinoma. Nevertheless, the diagnostic performance observed here is preliminary, as the study lacked external multi-center validation and only included one comparative cervical adenocarcinoma subtype. ARID1A loss cannot serve as an independent diagnostic marker but acts as a powerful auxiliary indicator when combined with morphological features and HPV testing.

### 3.5 Correlation between ARID1A status and clinicopathological characteristics of CCC

We stratified all CCC patients into ARID1A-negative (n = 14) and ARID1A-positive (n = 13) groups to compare the clinicopathological characteristics (**Table 3**). No significant inter-group differences were detected in age, tumor diameter, FIGO stage, lymph node metastasis, depth of cervical stromal infiltration, or recurrence/metastasis (P > 0.05). Consistent with the clinicopathological comparison, Kaplan–Meier survival curves revealed no significant differences in PFS and OS between the two groups (**Figure 7**). Conflicting prognostic associations of ARID1A deficiency have been documented across multiple solid tumors, but our cohort failed to capture any survival correlation specific to CCC. Given the limited number of recurrence and death events, and heterogeneous postoperative adjuvant treatment regimens across patients, we did not further explore ARID1A as a predictive biomarker for therapeutic response. Larger independent multi-center cohorts with standardized treatment protocols are required to confirm whether ARID1A deficiency bears subtle prognostic value in CCC.

**Table 3:** The correlation between ARID1A expression and the clinicopathological features of 27 patients with CCC.

Variable	Category	ARID1A not expressed (n = 14)	ARID1A expressed (n = 13)	*P* value
Age	≥40 years old	11	10	0.638
	<40 years old	3	3	
Tumor Diameter	≥4cm	4	7	0.173
	<4cm	10	6	
FIGO Staging	IA- IIA	11	10	0.638
	IIB-IV	3	3	
Lymph Node Metastasis	Present	1	2	0.471
	Absent	13	11	
Depth of Cervical Stromal Invasion	≥2/3 of the fibromuscular layer	7	7	0.573
	<2/3 of the fibromuscular layer	7	6	
Recurrence and Metastasis	Present	3	3	1.000
	Absent	11	10	

CCC, cervical clear cell carcinoma; FIGO, International Federation of Gynecology and Obstetrics. ARID1A loss was defined as complete absence of nuclear staining in tumor cells with preserved internal positive controls. Associations between ARID1A protein expression status and clinicopathological variables were evaluated using Fisher’s exact test or χ² test, as appropriate.

**Figure 7:**
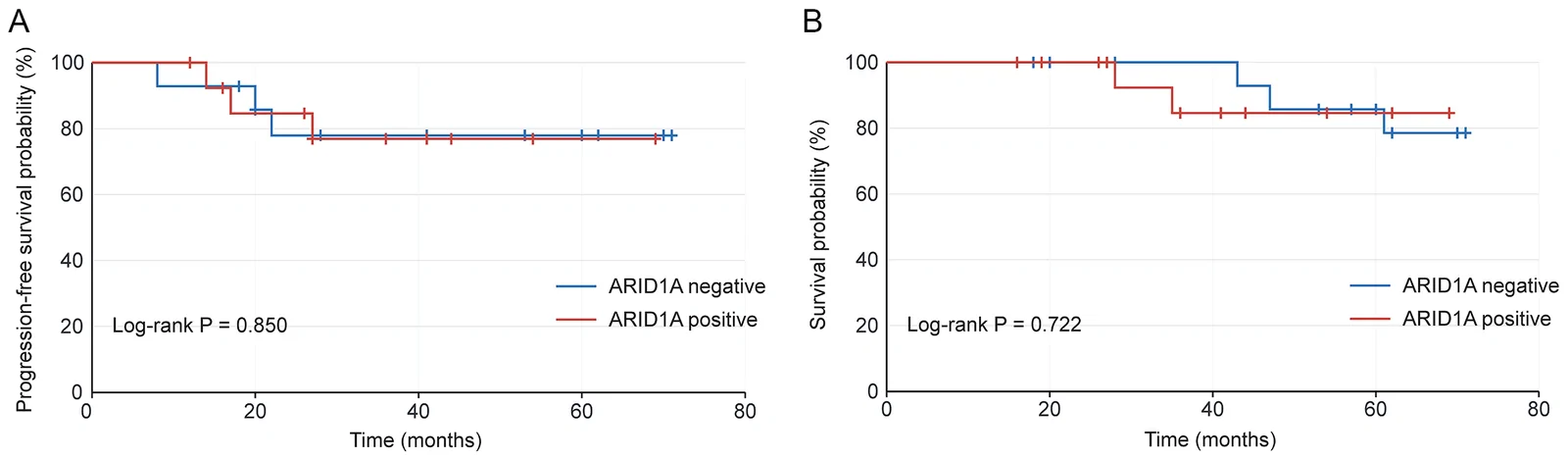
Kaplan–Meier curves of PFS and OS in CCC patients stratified by ARID1A expression. **(A, B)** Progression-free survival (PFS) and overall survival (OS) between CCC patients with negative and positive ARID1A tumor expression, respectively.

## 4 Discussion

Epidemiological research data indicate that approximately 15% of endocervical adenocarcinomas (ECA) lack HPV infection, representing a pathogenetically distinct disease subgroup (13). This non-HPV-associated subtype cannot benefit from HPV vaccine or traditional cervical exfoliated cytology screening. CCC belongs to this HPV-independent category, characterized by low overall incidence yet highly invasive biological behavior. Previous cohort studies have confirmed that CCC displays high susceptibility to lymph-vascular space invasion, lymph node metastasis, uterine corpus extension, and distant bone metastasis (4, 5). Stolnicu et al. analyzed 58 CCC patients and found that 72.4% of cases presented at FIGO stage I, with lymphovascular invasion occurring in 31% and lymph node metastasis in 24.1% of patients (6). Another retrospective analysis conducted by Thomas et al. identified lymph node status as the most strong predictor of OS and PFS (4).

In our study, the majority of patients were in the early stages of FIGO, with 22% experiencing recurrence or metastasis. In addition to FIGO staging, tumor size and pelvic lymph node status were identified as independent risk factors. Furthermore, it was observed that cancer tissue infiltration exceeding two-thirds of the fibromuscular layer, vaginal tissue involvement, parametrial infiltration, and distant metastasis of cancer tissue were also significant adverse prognostic factors. Considering that the overall rarity of CCC and limited published molecular and clinical data, our findings provide valuable real-world insights into this understudied gynecologic malignancy.

Targeted NGS identified concurrent somatic mutations across two SWI/SNF chromatin remodeling complexes, including SMARCA4 and ARID family members (ARID1A, ARID2) in three patients. GO analysis indicated significant enrichment of mutated genes in processes related to chromatin organization and remodeling. Approximately 20% of all human solid tumors harbor pathogenic mutations in SWI/SNF chromatin remodelers, rendering this complex one of the most frequently disrupted epigenetic regulatory machineries in oncogenesis (10). ARID1A represents the most frequently mutated subunit of the SWI/SNF chromatin remodeling complex across multiple human cancers (14, 15). Pathogenic ARID1A alterations occur in over 50% of ovarian clear cell carcinomas (11), 21% of gastric carcinomas (16), and 20% of colorectal malignancies (17). Our IHC results demonstrated complete ARID1A protein loss in 51.8% of CCC cases, consistent with prior findings in ovarian clear cell carcinoma with comparable morphological and immunophenotypic traits. This parallel observation suggests that recurrent ARID1A deficiency may constitute a shared molecular driver of clear cell carcinoma pathogenesis across female genital tract organs.

We further examined ARID1A loss frequency in HPVA as a comparative control subtype and found only 6.7% of HPVA specimens with ARID1A protein loss, significantly lower mutation rate compared to the CCC group. Within our study cohort, ARID1A loss showed a diagnostic sensitivity of 51.8% and a specificity of 93.3% for identifying CCC from HPVA. These estimates should be interpreted cautiously because they were derived from a relatively small, single-institution cohort without external validation. Therefore, ARID1A loss cannot serve as a potential adjunctive diagnostic marker but rather an auxiliary adjunct to routine pathological workup.

Our findings align with a prior independent cohort of 20 CCC patients analyzed by Kim et al., who identified pathogenic ARID1A mutations in 20% of cases via multi-platform sequencing (18). However, this previous study did not evaluate ARID1A protein loss via IHC or assess its diagnostic performance, precluding direct external validation of our IHC-based findings. From a translational laboratory perspective, ARID1A IHC can be seamlessly integrated into routine clinical pathology workflows using standard FFPE processing protocols. Complete loss of nuclear staining is relatively straightforward to interpret, making ARID1A a practical auxiliary marker for morphologically ambiguous CCC cases. Nevertheless, ARID1A loss is not entirely specific to CCC and should be interpreted in conjunction with histological features, HPV testing, and additional immunohistochemical markers to reach an accurate final diagnosis.

Recent studies have indicated that the loss of ARID1A function due to mutations is associated with increased PD-L1 expression and heightened tumor mutation burden (TMB) in ovarian and gastric cancers, implying enhanced anti-tumor immune surveillance in ARID1A-deficient tumors (19, 20). Previous preclinical studies have demonstrated that ARID1A-deficient malignancies exhibit heightened sensitivity to DNA damage-targeted therapies (PARP/ATR inhibitors) and immune checkpoint blockade. Despite these promising mechanistic observations, it remains unknown whether ARID1A deficiency predicts therapeutic response in CCC, as our study did not correlate ARID1A status with patient treatment outcomes. Existing clinical evidence supports combined immunotherapy for patients with ARID1A mutations (21), yet no ARID1A-targeted therapies are currently approved for CCC. The high prevalence ARID1A loss observed in our cohort provides a solid molecular rationale for future functional preclinical studies and biomarker-guided clinical investigation in CCC.

Given that HPV-positive cervical adenocarcinoma is driven by high-risk HPV oncoproteins (HPV16/18), viral dependency allows for the potential implementation of targeted treatment strategies based on viral presence, including vaccines and therapies targeting viral proteins (22). In contrast, CCC exhibits distinct molecular characteristics and likely develops through HPV-independent oncogenic pathways dominated by ARID1A inactivation. This fundamental molecular divergence underscores the clinical value of standardized HPV genotyping for subtyping cervical adenocarcinomas to guide prognosis and therapeutic selection.

Published literatures report conflicting prognostic associations for ARID1A loss across solid tumors. In gastric cancer, ARID1A mutation is positively associated with poor prognosis (23), while in colorectal and urothelial carcinoma ARID1A mutations may serve as a favorable prognostic factor (24, 25). In our CCC cohort, ARID1A protein loss showed no significant association with PFS or OS, nor with any standard clinicopathological variable. These negative findings indicate that ARID1A status cannot serve as an independent prognostic biomarker for CCC at present, and larger multi-center cohorts are required to validate this conclusion.

Accumulating evidence suggests associations of ARID1A loss with aberrant activation of PI3K signaling pathway across multiple malignancies. In gastric cancer, ARID1A loss directly triggers the activation of the PI3K/AKT/mTOR signaling pathway (26). In ovarian cancer cells harboring ARID1A mutations, PIK3IP1 expression is diminished, resulting in the activation of the PI3K/AKT/mTOR pathway (27). In ovarian clear cell carcinoma, ARID1A gene mutations cooperates with PIK3CA activation and PTEN loss to promote tumor progression via sustained PI3K/AKT pathway signaling (28). Additionally, Ueno et al. reported that 50% of CCC cases exhibited overexpression of both p-AKT and p-mTOR (9). The PI3K-AKT-mTOR pathway may contribute to CCC carcinogenesis by influencing cell proliferation, transformation and survival, highlighting it as a potential therapeutic target (29). KEGG enrichment analysis independently confirmed robust enrichment of mutated CCC genes within the PI3K-AKT signaling pathway. Previous studies in other tumor types have demonstrated that ARID1A loss can influence PI3K/AKT/mTOR pathway activity through mechanisms such as PTEN regulation and downstream signaling modulation. However, in the present study, we did not directly evaluate pathway activation using functional assays or protein-level analyses such as p-AKT, p-mTOR, or PTEN expression. Therefore, we only report a statistical association rather than definitive causal interaction between ARID1A loss and PI3K/AKT pathway activation in CCC, which requires dedicated *in vitro* and *in vivo* functional validation.

Lynch syndrome (LS) is predominantly associated with colorectal and endometrial cancer, though MMR variant carriers elevated risk of extra-uterine tumors across multiple organ sites (30). Following the first documented LS-associated CCC case reported by Nakamura et al. in 2018 (31), our research identified one CCC patient with a pathogenic germline MLH1 variant c.199G>A (p.G67R). This variant meets ACMG/AMP pathogenic criteria (PS3, PM2, PP3) due to its absence in gnomAD, computational predictions of deleteriousness (REVEL score = 0.92), and prior reports in Lynch syndrome. This patient presented no other LS-associated malignancies and had no clinical or histologic evidence of endometrial carcinoma. Although current evidence is insufficient to establish a definitive link between germline MMR mutations and cervical cancer, it is essential to conduct further research into the tendency of organs harboring MMR mutations to elevate cancer risk. This understanding will aid in identifying high-risk LS patients, assessing their risk of developing related cancers, and formulating appropriate risk management strategies.

Due to the rarity of CCC, this retrospective study enrolled 27 CCC patients, among whom only seven underwent targeted sequencing. Although our data offer meaningful molecular insights into this rare malignancy, several limitations should be noted. First, the small sample size restricts generalizability; our ARID1A immunohistochemistry results, which revealed higher ARID1A-loss frequency in CCC versus HPV-associated cervical adenocarcinoma, require external multi-cohort validation with broader histological comparators and standardized pathological assessment to define its true diagnostic utility. Second, our pathway analyses only indicated a statistical association between ARID1A alterations and PI3K-AKT-mTOR signaling, without direct functional validation. Further work using ARID1A-manipulated models and protein-level profiling (p-AKT, p-mTOR, PTEN) is needed to dissect their mechanistic crosstalk in CCC. Third, limited clinical events and the retrospective design precluded us from assessing whether ARID1A status predicts treatment response. Future prospective multicenter studies combining molecular profiling with detailed treatment records are warranted to evaluate the predictive biomarker potential of ARID1A deficiency.

## 5 Conclusion

CCC is a rare HPV-independent cervical adenocarcinoma characterized by distinct clinicopathological and molecular features. Our integrated genomic and immunohistochemical profiling demonstrates frequent ARID1A deficiency as a hallmark molecular alteration linked to global chromatin remodeling dysfunction and concurrent PI3K/AKT pathway genomic perturbations. Within this single-institution cohort, ARID1A loss occurs at substantially higher frequency in CCC compared with HPV-associated cervical adenocarcinoma, supporting its utility as a potential adjunctive diagnostic marker when combined with histological morphology, HPV testing, and complementary IHC biomarkers. Multi-center validation cohorts are required to confirm its diagnostic accuracy. Beyond its potential diagnostic applications, ARID1A deficiency constitutes a promising candidate biomarker to stratify patients for future therapeutic trials evaluating DNA damage response inhibitors, immune checkpoint blockade, and PI3K/AKT targeted agents. Collectively, these findings establish a molecular foundation for future biomarker-guided precision oncology research and therapeutic development for this rare, clinically challenging gynecologic malignancy.

## Data Availability

The original contributions presented in the study are included in the article/**Supplementary Material**. Further inquiries can be directed to the corresponding author.
