# Supplementary material for: ARID1A deficiency identifies molecular alterations and potential therapeutic implications in cervical clear cell carcinoma: an integrated genomic and clinicopathological study
Source: Front Oncol. 2026 Sep 21;16:1943760. doi: 10.3389/fonc.2026.1943760 (PMC13634973; doi:10.3389/fonc.2026.1943760)
Supplement: Supplementary file 1 [file Table1.docx]

**Supplementary files**

**Supplementary Table S1.** Targeted next-generation sequencing panel composition, including 312 cancer-related genes, fusion-related regions, and additional coding regions

1.1.1 312 Genes and All 4847 Exons Region

| *ABL1* | *ACVR1B* | *AKT1* | *AKT2* | *AKT3* | *ALK* | *APC* | *AR* | *ARAF* | *ARID1A* |
| --- | --- | --- | --- | --- | --- | --- | --- | --- | --- |
| *ARID1B* | *ARID2* | *ASXL1* | *ATM* | *ATR* | *ATRX* | *AURKA* | *AURKB* | *AXIN1* | *AXIN2* |
| *AXL* | *B2M* | *BAP1* | *BARD1* | *BCL2* | *BCL2L1* | *BCOR* | *BLM* | *BMPR1A* | *BRAF* |
| *BRCA1* | *BRCA2* | *BRD4* | *BRIP1* | *BTK* | *CARD11* | *CASP8* | *CBFB* | *CBL* | *CCND1* |
| *CCND2* | *CCND3* | *CCNE1* | *CD274* | *CDC73* | *CDH1* | *CDK12* | *CDK4* | *CDK6* | *CDK8* |
| *CDKN1A* | *CDKN1B* | *CDKN2A* | *CDKN2B* | *CDKN2C* | *CEBPA* | *CHEK1* | *CHEK2* | *CIC* | *CREBBP* |
| *CRKL* | *CSF1R* | *CTCF* | *CTNNA1* | *CTNNB1* | *CUL3* | *CYLD* | *DAXX* | *DDR1* | *DDR2* |
| *DICER1* | *DNMT3A* | *DOT1L* | *EGFR* | *EIF1AX* | *C11orf30* | *EP300* | *EPAS1* | *EPCAM* | *EPHA2* |
| *EPHA3* | *EPHA5* | *EPHB1* | *EPHB6* | *ERBB2* | *ERBB3* | *ERBB4* | *ERCC1* | *ERCC3* | *ERCC4* |
| *ERCC5* | *ERG* | *ERRFI1* | *ESR1* | *EXT1* | *EXT2* | *EZH2* | *FAM123B* | *FAM175A* | *FANCA* |
| *FANCC* | *FANCD2* | *FANCE* | *FANCF* | *FANCG* | *FANCL* | *FANCM* | *FAS* | *FAT1* | *FAT2* |
| *FBXW7* | *FGF19* | *FGF3* | *FGF4* | *FGFR1* | *FGFR2* | *FGFR3* | *FGFR4* | *FH* | *FLCN* |
| *FLT1* | *FLT3* | *FLT4* | *FOXA1* | *FOXL2* | *FOXP1* | *FUBP1* | *GALNT12* | *GATA3* | *GNA11* |
| *GNAQ* | *GNAS* | *GRIN2A* | *GRM3* | *HDAC1* | *HGF* | *HNF1A* | *HOXB13* | *HRAS* | *IDH1* |
| *IDH2* | *IFNG* | *IFNGR1* | *IGF1R* | *IKBKE* | *IKZF1* | *IL7R* | *INPP4B* | *IRF2* | *IRS2* |
| *JAK1* | *JAK2* | *JAK3* | *JUN* | *KDM5A* | *KDM5C* | *KDM6A* | *KDR* | *KEAP1* | *KIT* |
| *KRAS* | *LRP1B* | *MAF* | *MAP2K1* | *MAP2K2* | *MAP2K4* | *MAP3K1* | *MAPK1* | *MAX* | *MCL1* |
| *MDM2* | *MDM4* | *MED12* | *MEF2B* | *MEN1* | *MET* | *MITF* | *MLH1* | *MLH3* | *MLL* |
| *MLL2* | *MLL3* | *MPL* | *MRE11A* | *MS4A1* | *MSH2* | *MSH3* | *MSH6* | *MST1R* | *MTOR* |
| *MUTYH* | *MYC* | *MYCL1* | *MYCN* | *MYD88* | *NBN* | *NCOR1* | *NF1* | *NF2* | *NFE2L2* |
| *NFKBIA* | *NKX2-1* | *NOTCH1* | *NOTCH2* | *NOTCH3* | *NPM1* | *NRAS* | *NSD1* | *NTHL1* | *NTRK1* |
| *NTRK2* | *NTRK3* | *PALB2* | *PARK2* | *PARP1* | *PAX5* | *PBRM1* | *PCK1* | *PDCD1* | *PDCD1LG2* |
| *PDGFRA* | *PDGFRB* | *PDK1* | *PIK3CA* | *PIK3CB* | *PIK3CG* | *PIK3R1* | *PIK3R2* | *PMS1* | *PMS2* |
| *POLD1* | *POLE* | *POT1* | *PPP2R1A* | *PRDM1* | *PRKAR1A* | *PTCH1* | *PTCH2* | *PTEN* | *PTPN11* |
| *PTPRD* | *RAC1* | *RAD50* | *RAD51* | *RAD51B* | *RAD51C* | *RAD51D* | *RAD52* | *RAD54L* | *RAF1* |
| *RARA* | *RB1* | *RBM10* | *RECQL* | *RECQL4* | *RET* | *RHOA* | *RICTOR* | *RINT1* | *RNF43* |
| *ROS1* | *RPTOR* | *RUNX1* | *SDHA* | *SDHAF2* | *SDHB* | *SDHC* | *SDHD* | *SERPINB3* | *SERPINB4* |
| *SETD2* | *SF3B1* | *SLX4* | *SMAD2* | *SMAD3* | *SMAD4* | *SMARCA4* | *SMARCB1* | *SMO* | *SOCS1* |
| *SOX2* | *SOX9* | *SPOP* | *SRC* | *STAG2* | *STAT3* | *STK11* | *SUFU* | *SYK* | *TBX3* |
| *TCF7L2* | *TERC* | *TET2* | *TGFBR2* | *TMEM127* | *TMPRSS2* | *TNFAIP3* | *TNFRSF14* | *TOP1* | *TOP2A* |
| *TP53* | *TSC1* | *TSC2* | *TSHR* | *U2AF1* | *VEGFA* | *VHL* | *WRN* | *WT1* | *XPO1* |
| *XRCC2* | *ZMAT3* |  |  |  |  |  |  |  |  |

1.1.2 Regions Containing Introns, Promoters, and Fusion Breakpoints Across 38 Genes

| *ALK* | *BCL2L11* | *BRAF* | *BRCA1* | *BRD4* | *CD74* | *EGFR* | *EML4* | *ERG* | *ETV6* |
| --- | --- | --- | --- | --- | --- | --- | --- | --- | --- |
| *EZR* | *FGFR1* | *FGFR2* | *FGFR3* | *KIF5B* | *KIT* | *MAML2* | *MET* | *MSH2* | *MYC* |
| *MYCL1* | *NCOA4* | *NOTCH2* | *NTRK1* | *NTRK2* | *NTRK3* | *PDGFRA* | *RAF1* | *RET* | *ROS1* |
| *RSPO2* | *SDC4* | *SLC34A2* | *TERT* | *TFE3* | *TMPRSS2* | *TPM3* | *PMS2* |  |  |

1.1.3 1,778 Coding Regions Spanning 709 Other Related Genes

| *ABCA13* | *ABCB1* | *ABCC1* | *ABCC11* | *ABCC2* | *ABCG2* | *ABL2* | *ACACA* | *ACIN1* | *ACTB* |
| --- | --- | --- | --- | --- | --- | --- | --- | --- | --- |
| *ACTG1* | *ACTG2* | *ACVR2A* | *ACVRL1* | *ADAM29* | *ADAMTS5* | *ADCY1* | *AFF1* | *AFF2* | *AFF3* |
| *AHNAK* | *AKAP9* | *ALB* | *AMOT* | *ANGPT1* | *ANK3* | *ANKRD11* | *ANKRD30A* | *ANKRD30B* | *APEX1* |
| *APOBEC3B* | *ARAP3* | *ARFGEF1* | *ARFGEF2* | *ARHGAP29* | *ARHGAP35* | *ARID4B* | *ARID5B* | *ARNT* | *ASCL4* |
| *ASH1L* | *ASMTL* | *ASPM* | *ASTN1* | *ASXL2* | *ATIC* | *ATP11B* | *ATP12A* | *ATP1A1* | *ATP2B3* |
| *BAZ2B* | *BBC3* | *BBS9* | *BCAS1* | *BCL10* | *BCL11A* | *BCL11B* | *BCL2A1* | *BCL2L11* | *BCL3* |
| *BCL6* | *BCL9* | *BCORL1* | *BCR* | *BIRC3* | *BMPR2* | *BNC2* | *BPTF* | *BRD2* | *BRD3* |
| *BRSK1* | *BRWD1* | *BTLA* | *BUB1* | *C15orf23* | *C15orf55* | *C1QA* | *C1S* | *C3orf70* | *C7orf53* |
| *C8orf34* | *CACNA1E* | *CADM2* | *CALR* | *CAMTA1* | *CASP1* | *CASQ2* | *CBLB* | *CBR1* | *CBR3* |
| *CCDC168* | *CCNA1* | *CCNB3* | *CCT3* | *CCT5* | *CCT6B* | *CD22* | *CD33* | *CD5L* | *CD74* |
| *CDA* | *CDH11* | *CDH18* | *CDH23* | *CDK13* | *CHD1* | *CHD1L* | *CHD4* | *CHD6* | *CHD8* |
| *CHD9* | *CHFR* | *CHI3L1* | *CHN1* | *CIITA* | *CLDN18* | *CLP1* | *CLSPN* | *CLTC* | *CNOT3* |
| *CNOT4* | *CNTN1* | *CNTN5* | *CNTNAP1* | *CNTNAP5* | *COL1A1* | *COL2A1* | *COL5A1* | *COL5A2* | *COL5A3* |
| *COPS2* | *CPS1* | *CRIPAK* | *CRLF2* | *CRNKL1* | *CRTC1* | *CSF1* | *CSF3R* | *CSMD1* | *CSMD3* |
| *CSNK1A1* | *CSNK1G3* | *CTLA4* | *CTNNA2* | *CTNND1* | *CUX1* | *CXCR4* | *CYBA* | *CYP19A1* | *CYP1A1* |
| *CYP1B1* | *CYP2A13* | *CYP2C8* | *CYP2D6* | *CYP3A4* | *CYP3A5* | *DCC* | *DDX3X* | *DDX5* | *DEK* |
| *DHX35* | *DHX9* | *DIAPH1* | *DIS3L2* | *DLC1* | *DMD* | *DNAH6* | *DNAJB1* | *DNM2* | *DNMT1* |
| *DNMT3B* | *DOCK2* | *DOCK7* | *DPYD* | *DRGX* | *DTX1* | *DUSP22* | *DYSF* | *E2F3* | *EBF1* |
| *ECT2L* | *EED* | *EEF1A1* | *EGFL7* | *EGR3* | *EIF2AK3* | *EIF2C3* | *EIF3A* | *EIF4A2* | *EIF4G3* |
| *ELAC2* | *ELF1* | *ELF3* | *ELMO1* | *ELN* | *EME2* | *EMID2* | *EML4* | *EPC1* | *EPHA1* |
| *EPHA4* | *EPHA7* | *EPHB2* | *EPHB4* | *EPOR* | *EPPK1* | *EPS15* | *ERBB2IP* | *ERCC2* | *ESR2* |
| *ETS1* | *ETV1* | *ETV5* | *ETV6* | *EWSR1* | *EZR* | *F8* | *FAM131B* | *FAM135B* | *FAM157B* |
| *FAM46C* | *FAM5C* | *FAP* | *FASLG* | *FAT3* | *FAT4* | *FCGR1A* | *FCGR2A* | *FCGR2B* | *FCGR3A* |
| *FCRL4* | *FGF10* | *FGF12* | *FGF14* | *FGF23* | *FGF6* | *FLG* | *FLI1* | *FLNC* | *FMN2* |
| *FN1* | *FNDC4* | *FOXA2* | *FOXO1* | *FOXO3* | *FOXQ1* | *FRMPD4* | *FUS* | *FXR1* | *FYN* |
| *FZD1* | *G3BP1* | *G3BP2* | *GAB2* | *GABRA6* | *GATA1* | *GATA2* | *GFRAL* | *GIGYF1* | *GKN2* |
| *GLB1L3* | *GLI1* | *GLI2* | *GLI3* | *GMPS* | *GNA13* | *GNG2* | *GPC3* | *GPR124* | *GPS2* |
| *GPX1* | *GRB7* | *GSK3B* | *GSTM5* | *GSTP1* | *GUSB* | *H3F3A* | *H3F3B* | *H3F3C* | *HCLS1* |
| *HCN1* | *HDAC4* | *HDAC9* | *HECW1* | *HEY1* | *HIST1H1C* | *HIST1H1D* | *HIST1H1E* | *HIST1H2AC* | *HIST1H2AG* |
| *HIST1H2AL* | *HIST1H2AM* | *HIST1H2BC* | *HIST1H2BD* | *HIST1H2BJ* | *HIST1H2BK* | *HIST1H2BO* | *HIST1H3B* | *HIST1H3C* | *HIST1H3D* |
| *HIST1H3F* | *HIST1H3G* | *HIST1H3H* | *HIST1H3I* | *HIST1H4I* | *HIST3H3* | *HLA-A* | *HLA-B* | *HLA-C* | *HLF* |
| *HMCN1* | *HNF1B* | *HNRPDL* | *HOXA11* | *HOXA13* | *HOXA3* | *HOXA9* | *HOXC13* | *HOXD11* | *HOXD13* |
| *HSD3B1* | *HSP90AA1* | *HSP90AB1* | *HSPA8* | *HSPD1* | *HSPH1* | *ICK* | *ICOSLG* | *ID3* | *IFITM3* |
| *IGF1* | *IGF2* | *IGF2R* | *IGLL5* | *IKZF2* | *IKZF3* | *IL10* | *IL1RAPL1* | *IL21R* | *IL6* |
| *IL6ST* | *IMPG1* | *ING1* | *INHBA* | *INPP4A* | *INPPL1* | *INSR* | *IRF4* | *IRF6* | *IRS1* |
| *ITGB3* | *ITK* | *ITSN1* | *JARID2* | *KALRN* | *KAT6A* | *KAT6B* | *KCNJ5* | *KCNQ2* | *KDM2B* |
| *KEL* | *KIF5B* | *KLF4* | *KLHL6* | *KLK1* | *KRTAP5-5* | *L3MBTL1* | *LAMA2* | *LATS1* | *LATS2* |
| *LCP1* | *LEF1* | *LGALS8* | *LIFR* | *LPHN2* | *LPP* | *LRP2* | *LRP4* | *LRP5* | *LRP6* |
| *LRRC7* | *LRRK2* | *LYN* | *LZTS1* | *MACF1* | *MAD1L1* | *MAGI2* | *MAML2* | *MAML3* | *MAP3K13* |
| *MAPK3* | *MCC* | *MCM3* | *MDC1* | *MECOM* | *MEF2C* | *MGA* | *MIB1* | *MIOS* | *MKL1* |
| *MLL4* | *MLLT3* | *MMP11* | *MMP2* | *MN1* | *MNDA* | *MNX1* | *MSH4* | *MSN* | *MSR1* |
| *MTHFR* | *MTRR* | *MUC5B* | *MYH11* | *MYH14* | *MYH9* | *MYO3A* | *MYOD1* | *NAP1L1* | *NAV3* |
| *NCAM2* | *NCF2* | *NCF4* | *NCK1* | *NCOA3* | *NCOA4* | *NCOR2* | *NCSTN* | *NDUFA13* | *NFATC4* |
| *NFE2L3* | *NKX3-1* | *NLRC3* | *NOD1* | *NOS3* | *NOTCH4* | *NQO1* | *NR1I2* | *NR2F2* | *NR4A2* |
| *NRG1* | *NRP2* | *NRXN1* | *NTM* | *NUMA1* | *NUP107* | *NUP210* | *NUP93* | *NUP98* | *OBSCN* |
| *OGDH* | *OMD* | *OPCML* | *OR11G2* | *OR2T4* | *OR4A15* | *OR4C6* | *OR5L2* | *OR6F1* | *P2RY8* |
| *P4HB* | *PABPC1* | *PABPC3* | *PAG1* | *PAK1* | *PAK3* | *PASK* | *PAX3* | *PAX7* | *PC* |
| *PCDH18* | *PCSK6* | *PCSK7* | *PDCD11* | *PDE4DIP* | *PDGFB* | *PDILT* | *PER1* | *PGR* | *PHF1* |
| *PHF6* | *PIK3C2A* | *PIK3C2B* | *PIK3C2G* | *PIK3C3* | *PIM1* | *PKD1L2* | *PKHD1* | *PLAG1* | *PLCB1* |
| *PLCG1* | *PLCG2* | *PLK1* | *PLXNA1* | *PLXNB2* | *PNRC1* | *POLQ* | *POM121* | *POM121L12* | *POU2AF1* |
| *PPM1D* | *PPP1R17* | *PPP6C* | *PRDM16* | *PREX2* | *PRF1* | *PRKAA1* | *PRKCB* | *PRKCI* | *PRKDC* |
| *PRRX1* | *PRX* | *PSG2* | *PSIP1* | *PSMB1* | *PSMB5* | *PTGS1* | *PTGS2* | *PTPN13* | *PTPN2* |
| *PTPRB* | *PTPRK* | *PTPRO* | *PTPRS* | *PTPRT* | *PTPRU* | *RAB35* | *RAC2* | *RAD21* | *RAD54B* |
| *RANBP2* | *RASA1* | *RASGRP1* | *RBL1* | *REL* | *RELN* | *RFC1* | *RGS3* | *RHEB* | *RHOH* |
| *RHOT1* | *RIT1* | *RNASEL* | *ROBO1* | *ROBO2* | *ROBO3* | *ROCK1* | *RPGR* | *RPS6KB1* | *RPS6KB2* |
| *RSPO2* | *RSPO3* | *RUNX1T1* | *RUNX2* | *RXRA* | *RYR1* | *RYR2* | *SBDS* | *SCUBE2* | *SDC4* |
| *SEC31A* | *SEMA3A* | *SEMA3E* | *SEMA6A* | *SERPINA7* | *SETBP1* | *SETDB1* | *SF1* | *SF3A1* | *SFPQ* |
| *SGCZ* | *SGK1* | *SH2B3* | *SH2D1A* | *SH3PXD2A* | *SHH* | *SI* | *SIN3A* | *SLC16A1* | *SLC1A2* |
| *SLC22A16* | *SLC22A18* | *SLC22A2* | *SLC22A3* | *SLC34A2* | *SLCO1B3* | *SLIT1* | *SLIT2* | *SMARCD1* | *SMARCE1* |
| *SMC1A* | *SMC1B* | *SNCAIP* | *SNTG1* | *SNX29* | *SOD2* | *SOS1* | *SOX10* | *SOX17* | *SPEN* |
| *SPRR3* | *SPSB4* | *SPTA1* | *SRD5A2* | *SRGAP1* | *SRGAP3* | *SRSF2* | *SRSF7* | *STAG1* | *STAT1* |
| *SUCLG1* | *SUCLG2* | *SULT1A1* | *SUZ12* | *SVEP1* | *SYNCRIP* | *SYNE1* | *TAF1* | *TAF15* | *TAF1L* |
| *TAL1* | *TBL1XR1* | *TBX15* | *TBX22* | *TCEB1* | *TCF12* | *TCF3* | *TCF4* | *TCL1A* | *TEC* |
| *TENM3* | *TERT* | *TET1* | *TFDP1* | *TFDP2* | *TFE3* | *TGFBR1* | *THBS2* | *TJP1* | *TLE1* |
| *TLL2* | *TLR4* | *TLX3* | *TMEM132D* | *TNFSF11* | *TNN* | *TP53BP1* | *TP63* | *TP73* | *TPM3* |
| *TPR* | *TRAF2* | *TRAF7* | *TRIM24* | *TRIM58* | *TRIO* | *TRPC5* | *TRRAP* | *TSHZ2* | *TSHZ3* |
| *TTF1* | *TUBA3C* | *TUBB3* | *TUSC3* | *TXNIP* | *TYMS* | *TYR* | *UBE2D2* | *UBR5* | *UGT1A1* |
| *UMPS* | *UPF3B* | *USH2A* | *USP6* | *USP8* | *VEZF1* | *VIM* | *VTCN1* | *WASF3* | *WDR90* |
| *WDTC1* | *WHSC1* | *WHSC1L1* | *WIPF1* | *WNK1* | *WNT5A* | *WSCD2* | *WWOX* | *WWP1* | *WWP2* |
| *XIAP* | *XPC* | *XRCC1* | *XRCC3* | *YAP1* | *YY1AP1* | *ZBTB16* | *ZC3H11A* | *ZFHX3* | *ZFP36L1* |
| *ZFP36L2* | *ZFPM2* | *ZIC3* | *ZNF217* | *ZNF384* | *ZNF521* | *ZNF638* | *ZNF750* | *ZNF804B* |  |

1.1.4 Germline Variant Gene Detection List
1.1.5 Full Exons of 36 Genes

| *APC* | *ATM* | *ATRX* | *BAP1* | *BARD1* | *BLM* | *BRCA1* | *BRCA2* | *BRIP1* | *CDK12* |
| --- | --- | --- | --- | --- | --- | --- | --- | --- | --- |
| *CHEK2* | *ERCC1* | *FANCA* | *FANCC* | *FANCD2* | *FANCE* | *FANCF* | *FANCG* | *FANCL* | *FANCM* |
| *MLH1* | *MRE11A* | *MSH2* | *MSH6* | *NBN* | *PALB2* | *PMS2* | *RAD50* | *RAD51* | *RAD51B* |
| *RAD51C* | *RAD51D* | *RAD54L* | *RECQL* | *RECQL4* | *WRN* |  |  |  |  |

**Supplementary Table S2**. Distribution of somatic alterations identified in individual CCC samples

| **Sample** | **date** | **TMB status** | **TMB value** | **TMB percentile** | **Category** | **Gene** | **Transcript** | **Protein variant** | **Exon** | **VAF** |
| --- | --- | --- | --- | --- | --- | --- | --- | --- | --- | --- |
| S1 | 2022/10/31 | TMB-L | 4.8 | 57% | SNV/indel | SPOP |  | p.R121G |  | 29.50% |
| S1 | 2022/10/31 | TMB-L | 4.8 | 57% | SNV/indel | RPTOR |  | p.M511 |  | 17.50% |
| S1 | 2022/10/31 | TMB-L | 4.8 | 57% | SNV/indel | PPP2R1A |  | p.R183W |  | 17.20% |
| S1 | 2022/10/31 | TMB-L | 4.8 | 57% | SNV/indel | EPHA2 |  | p.V454D |  | 17.10% |
| S1 | 2022/10/31 | TMB-L | 4.8 | 57% | SNV/indel | PIK3CA |  | p.Q546R | EX10 | 16.10% |
| S1 | 2022/10/31 | TMB-L | 4.8 | 57% | SNV/indel | ARID1A |  | p.O372Sfs*19 |  | 14.80% |
| S1 | 2022/10/31 | TMB-L | 4.8 | 57% | SNV/indel | ERCC5 |  | p.R138* |  | 3.70% |
| S1 | 2022/10/31 | TMB-L | 4.8 | 57% | SNV/indel | CDC73 |  | p.V146A |  | 1.30% |
| S2 | 2019/9/24 | TMB-L | 2.88 | 15% | SNV/indel | EPHA3 |  | p.N884K | EX15 | 20.00% |
| S2 | 2019/9/24 | TMB-L | 2.88 | 15% | SNV/indel | SMAD4 |  | p.R361H |  | 12.10% |
| S2 | 2019/9/24 | TMB-L | 2.88 | 15% | SNV/indel | ABL1 |  | p.G7065 | EX11 | 5.30% |
| S2 | 2019/9/24 | TMB-L | 2.88 | 15% | SNV/indel | JAK3 |  | D.A306V |  | 1.90% |
| S2 | 2019/9/24 | TMB-L | 2.88 | 15% | CNV | NA | NM_000245.2 |  | all exon |  |
| S3 | 2020.3-30 | TMB-L | 5.76 | 56% | SNV/indel | EPHA3 |  | p.N884K | EX15 | 31.00% |
| S3 | 2020.3-30 | TMB-L | 5.76 | 56% | SNV/indel | PRF1 |  | p.E281del |  | 13.30% |
| S3 | 2020.3-30 | TMB-L | 5.76 | 56% | Intronic/splice-region variant | EGFR |  | IVS6 |  | 7.40% |
| S3 | 2020.3-30 | TMB-L | 5.76 | 56% | SNV/indel | ARID2 |  | p.Q1096* | EX15 | 6.20% |
| S3 | 2020.3-30 | TMB-L | 5.76 | 56% | SNV/indel | NCQR1 |  | p.D146Y | EX5 | 4.20% |
| S3 | 2020.3-30 | TMB-L | 5.76 | 56% | SNV/indel | FGFR3 |  | p.R421* | EX9 | 4.00% |
| S3 | 2020.3-30 | TMB-L | 5.76 | 56% | SNV/indel | KDM5A |  | p.R1417M | EX26 | 2.00% |
| S3 | 2020.3-30 | TMB-L | 5.76 | 56% | CNV | NA | NM_000245.2 |  | all exon |  |
| S4 | 2023/3/3 | TMB-L | 1.92 | 20% | SNV/indel | SMARCA4 | NM 003072.3 | p.Q1440* | EX30 | 3.20% |
| S4 | 2023/3/3 | TMB-L | 1.92 | 20% | SNV/indel | SMARCA4 |  | p.A791V | EX16 | 2.10% |
| S5 | 2023/6/13 | TMB-L |  |  | SNV/indel | NTRK2 |  | p.I289V | EX11 | 22.76% |
| S5 | 2023/6/13 | TMB-L |  |  | Fusion/rearrangement | UBN1-FOXL2 |  | IVS7_EX1 |  | 38.51% |
| S5 | 2023/6/13 | TMB-L |  |  | CNV | ZNF217 |  |  | 20q13.2; all exon |  |
| S6 | 2021/8/16 | TMB-H | 53.76 | 99% | SNV/indel | NA |  | p.L15Ffs*41 |  | 27.90% |
| S6 | 2021/8/16 | TMB-H | 53.76 | 99% | SNV/indel | MLL3 |  | p.K2797Rfs*26 | EX38 | 22.00% |
| S6 | 2021/8/16 | TMB-H | 53.76 | 99% | SNV/indel | SMARCA4 |  | p.T894M | EX19 | 21.80% |
| S6 | 2021/8/16 | TMB-H | 53.76 | 99% | SNV/indel | PTCH2 |  | p.A997V | EX19 | 21.50% |
| S6 | 2021/8/16 | TMB-H | 53.76 | 99% | SNV/indel | RBM10 |  | P.R725H | EX18 | 21.50% |
| S6 | 2021/8/16 | TMB-H | 53.76 | 99% | SNV/indel | GSK3B |  | p.G34D |  | 21.40% |
| S6 | 2021/8/16 | TMB-H | 53.76 | 99% | SNV/indel | PTEN |  | p.K267Rfs*9 |  | 21.30% |
| S6 | 2021/8/16 | TMB-H | 53.76 | 99% | SNV/indel | MLL2 |  | p.Q827Nfs*103 | FX10 | 21.30% |
| S6 | 2021/8/16 | TMB-H | 53.76 | 99% | SNV/indel | FGFR3 |  | p.A569W | EX13 | 21.20% |
| S6 | 2021/8/16 | TMB-H | 53.76 | 99% | SNV/indel | TRRAP |  | p.P698S | EX18 | 20.70% |
| S6 | 2021/8/16 | TMB-H | 53.76 | 99% | SNV/indel | RAD54L |  | p.R106C |  | 20.30% |
| S6 | 2021/8/16 | TMB-H | 53.76 | 99% | SNV/indel | BCL3 |  | p.R409Gfs*73 |  | 20.30% |
| S6 | 2021/8/16 | TMB-H | 53.76 | 99% | SNV/indel | RECQL4 |  | p.V364M |  | 20.10% |
| S6 | 2021/8/16 | TMB-H | 53.76 | 99% | Intronic/splice-region variant | CNTN1 |  | IVS2] |  | 20.10% |
| S6 | 2021/8/16 | TMB-H | 53.76 | 99% | SNV/indel | DICER1 |  | p.R392Q |  | 20.00% |
| S6 | 2021/8/16 | TMB-H | 53.76 | 99% | SNV/indel | DIS3L2 |  | p.D626N | EX15 | 19.90% |
| S6 | 2021/8/16 | TMB-H | 53.76 | 99% | SNV/indel | ABL1 |  | p.R617C | EX11 | 19.90% |
| S6 | 2021/8/16 | TMB-H | 53.76 | 99% | SNV/indel | NOTCHI |  | p.A2463T | EX34 | 19.90% |
| S6 | 2021/8/16 | TMB-H | 53.76 | 99% | SNV/indel | CYLD |  | p.174Ffs*2 |  | 19.80% |
| S6 | 2021/8/16 | TMB-H | 53.76 | 99% | SNV/indel | FOXP1 |  | p.Q114* |  | 19.70% |
| S6 | 2021/8/16 | TMB-H | 53.76 | 99% | SNV/indel | HNF1A |  | p.P379Lfs*5 |  | 19.50% |
| S6 | 2021/8/16 | TMB-H | 53.76 | 99% | SNV/indel | MLL4 |  | p.P1101Lfs*81 |  | 19.50% |
| S6 | 2021/8/16 | TMB-H | 53.76 | 99% | SNV/indel | MYCL1 |  | p.R324W |  | 19.40% |
| S6 | 2021/8/16 | TMB-H | 53.76 | 99% | SNV/indel | CTCF |  | p.T204Qfs*18 |  | 19.40% |
| S6 | 2021/8/16 | TMB-H | 53.76 | 99% | SNV/indel | FAT1 |  | p.R4160H | EX25 | 19.20% |
| S6 | 2021/8/16 | TMB-H | 53.76 | 99% | SNV/indel | BCL2 |  | p.A45Hfs*51 |  | 19.20% |
| S6 | 2021/8/16 | TMB-H | 53.76 | 99% | SNV/indel | LRP1B |  | p.T3295Pfs*118 | EX62 | 18.90% |
| S6 | 2021/8/16 | TMB-H | 53.76 | 99% | SNV/indel | FAT1 |  | P.Q1494* |  | 18.80% |
| S6 | 2021/8/16 | TMB-H | 53.76 | 99% | SNV/indel | ASXL1 |  | p.G645Vfs*58 | EX13 | 18.80% |
| S6 | 2021/8/16 | TMB-H | 53.76 | 99% | SNV/indel | BAP1 |  | p.0? |  | 18.60% |
| S6 | 2021/8/16 | TMB-H | 53.76 | 99% | SNV/indel | IL6ST |  | p.V530*fs*1 | EX13 | 18.20% |
| S6 | 2021/8/16 | TMB-H | 53.76 | 99% | SNV/indel | ZFHX3 |  | p.A3407Lfs*78 | EX10 | 18.20% |
| S6 | 2021/8/16 | TMB-H | 53.76 | 99% | SNV/indel | DNMZ |  | p.L793Pfs*31 | EX20 | 18.20% |
| S6 | 2021/8/16 | TMB-H | 53.76 | 99% | SNV/indel | NA |  | p.51245Afs*57 | EX15 | 18.20% |
| S6 | 2021/8/16 | TMB-H | 53.76 | 99% | SNV/indel | PTEN |  | p.N323Rfs*3 |  | 18.00% |
| S6 | 2021/8/16 | TMB-H | 53.76 | 99% | SNV/indel | MAP2K4 |  | p.P23T | EX1 | 18.00% |
| S6 | 2021/8/16 | TMB-H | 53.76 | 99% | SNV/indel | GNAS |  | p.R201H |  | 18.00% |
| S6 | 2021/8/16 | TMB-H | 53.76 | 99% | SNV/indel | NOTCH1 |  | p.S1690L | EX27 | 17.90% |
| S6 | 2021/8/16 | TMB-H | 53.76 | 99% | SNV/indel | ARID1A |  | p.Q1519Rfs*8 | EX18 | 17.50% |
| S6 | 2021/8/16 | TMB-H | 53.76 | 99% | SNV/indel | FGFR4 |  | p.H65Y |  | 17.20% |
| S6 | 2021/8/16 | TMB-H | 53.76 | 99% | Intronic/splice-region variant | RET |  | IVS9 |  | 17.00% |
| S6 | 2021/8/16 | TMB-H | 53.76 | 99% | SNV/indel | HDAC4 |  | p.M902Wfs*55 | EX22 | 16.80% |
| S6 | 2021/8/16 | TMB-H | 53.76 | 99% | SNV/indel | ARID1A |  | p.D1850Tfs*33 | EX20 | 16.70% |
| S6 | 2021/8/16 | TMB-H | 53.76 | 99% | SNV/indel | TOP2A |  | p.K344del |  | 16.60% |
| S6 | 2021/8/16 | TMB-H | 53.76 | 99% | SNV/indel | TSC1 |  | p.51043del | EX23 | 16.40% |
| S6 | 2021/8/16 | TMB-H | 53.76 | 99% | SNV/indel | MAP3K1 |  | p.P971Ffs*32 | EX14 | 16.30% |
| S6 | 2021/8/16 | TMB-H | 53.76 | 99% | SNV/indel | BCORL1 |  | p.P1681Qfs*20 | EX12 | 15.40% |
| S6 | 2021/8/16 | TMB-H | 53.76 | 99% | SNV/indel | FBXW7 |  | p.T463N |  | 14.10% |
| S6 | 2021/8/16 | TMB-H | 53.76 | 99% | SNV/indel | NA |  | p.R1445del | EX24 | 13.50% |
| S6 | 2021/8/16 | TMB-H | 53.76 | 99% | SNV/indel | POM121 |  | p.A430_K436del | EX14 | 12.30% |
| S6 | 2021/8/16 | TMB-H | 53.76 | 99% | Intronic/splice-region variant | PBRM1 |  | IVS17 |  | 10.50% |
| S6 | 2021/8/16 | TMB-H | 53.76 | 99% | SNV/indel | TRAF2 |  | p.K140N |  | 9.80% |
| S6 | 2021/8/16 | TMB-H | 53.76 | 99% | SNV/indel | SMARCA4 |  | p.S46Afs*48 |  | 7.20% |
| S6 | 2021/8/16 | TMB-H | 53.76 | 99% | SNV/indel | CTNNB1 |  | p.C350Y |  | 5.80% |
| S6 | 2021/8/16 | TMB-H | 53.76 | 99% | SNV/indel | ATRX |  | D.P5H |  | 5.60% |
| S6 | 2021/8/16 | TMB-H | 53.76 | 99% | SNV/indel | ESR1 |  | p.G77D |  | 5.40% |
| S6 | 2021/8/16 | TMB-H | 53.76 | 99% | SNV/indel | JAK3 |  | p.S340Vfs*69 |  | 2.50% |
| S6 | 2021/8/16 | TMB-H | 53.76 | 99% | SNV/indel | CHEK2 |  | p.A215T |  | 2.20% |
| S6 | 2021/8/16 | TMB-H | 53.76 | 99% | SNV/indel | FLT4 |  | p.L176M |  | 2.10% |
| S6 | 2021/8/16 | TMB-H | 53.76 | 99% | SNV/indel | HISTIH3B |  | p.P67S |  | 2.00% |
| S6 | 2021/8/16 | TMB-H | 53.76 | 99% | SNV/indel | NDUFA13 |  | p.K5E |  | 1.80% |
| S6 | 2021/8/16 | TMB-H | 53.76 | 99% | SNV/indel | BRCA2 |  | P.A1981S | EX11 | 1.00% |
| S6 | 2021/8/16 | TMB-H | 53.76 | 99% | SNV/indel | DOT1L |  | p.T735M | EX20 | 1.00% |
| S6 | 2021/8/16 | TMB-H | 53.76 | 99% | Germline variant | MLHI | NM_000249.3 | c.199G>A; p.G67R |  |  |
